# RNA Is a Double-Edged Sword in ALS Pathogenesis

**DOI:** 10.3389/fncel.2021.708181

**Published:** 2021-07-19

**Authors:** Benjamin L. Zaepfel, Jeffrey D. Rothstein

**Affiliations:** ^1^Biochemistry, Cellular and Molecular Biology Program, Johns Hopkins University School of Medicine, Baltimore, MD, United States; ^2^Molecular Biology and Genetics Department, Johns Hopkins University School of Medicine, Baltimore, MD, United States; ^3^Brain Science Institute, Johns Hopkins University School of Medicine, Baltimore, MD, United States; ^4^Department of Neurology, Johns Hopkins University School of Medicine, Baltimore, MD, United States

**Keywords:** amyotrophic lateral sclerosis, TDP43, FTD, FUS, RNA, C9ORF72 ALS/FTD

## Abstract

Amyotrophic lateral sclerosis (ALS) is a progressive and fatal neurodegenerative disease that affects upper and lower motor neurons. Familial ALS accounts for a small subset of cases (<10–15%) and is caused by dominant mutations in one of more than 10 known genes. Multiple genes have been causally or pathologically linked to both ALS and frontotemporal dementia (FTD). Many of these genes encode RNA-binding proteins, so the role of dysregulated RNA metabolism in neurodegeneration is being actively investigated. In addition to defects in RNA metabolism, recent studies provide emerging evidence into how RNA itself can contribute to the degeneration of both motor and cortical neurons. In this review, we discuss the roles of altered RNA metabolism and RNA-mediated toxicity in the context of *TARDBP, FUS*, and *C9ORF72* mutations. Specifically, we focus on recent studies that describe toxic RNA as the potential initiator of disease, disease-associated defects in specific RNA metabolism pathways, as well as how RNA-based approaches can be used as potential therapies. Altogether, we highlight the importance of RNA-based investigations into the molecular progression of ALS, as well as the need for RNA-dependent structural studies of disease-linked RNA-binding proteins to identify clear therapeutic targets.

## Introduction

Since the discovery of mutations in *TARDBP*, *FUS*, and *C9ORF72* that cause amyotrophic lateral sclerosis (ALS) and frontotemporal dementia (FTD), numerous investigations have demonstrated the effect these mutations have on RNA metabolism, as well as how RNA itself contributes to pathogenicity (Tollervey et al., 2011; Xiao et al., 2011; Daigle et al., 2013; Donnelly et al., 2013; Gendron et al., 2013; Ihara et al., 2013; Lagier-Tourenne et al., 2013; Lee et al., 2013; Nakaya et al., 2013; Shodai et al., 2013; Cooper-Knock et al., 2014; Coyne et al., 2014, 2015, 2017a, 2020; Highley et al., 2014; Liu-Yesucevitz et al., 2014; Mackness et al., 2014; Barmada et al., 2015; Burguete et al., 2015; Tran et al., 2015; Conlon et al., 2016; Dodd et al., 2016; Garnier et al., 2017; Liu et al., 2017; Lu et al., 2017; Kamelgarn et al., 2018; Tank et al., 2018; Chen et al., 2019; Xu et al., 2019; Ortega et al., 2020; Sun et al., 2020; Zaepfel et al., 2021). Mutations in all three of these genes have been linked to alterations in mRNA splicing and stress granule formation (Daigle et al., 2013; Nakaya et al., 2013; Conlon et al., 2016; Donde et al., 2019; Klim et al., 2019). However, we do not discuss such defects herein. Here, we discuss a bulk of the findings where direct or indirect alterations in RNA-related pathways have been linked to mutations in these three genes. We also address the role of RNA itself in mediating this toxicity. With a focus on the structure of proteins as they relate to RNA-binding and -processing functions, we provide new insight into the bi-directional effect between RNA itself and altered RNA processing in ALS.

## TDP-43

### Introduction

*TARDBP* encodes the TAR DNA-binding protein (TDP-43), which was initially discovered as a protein that binds pyrimidine-rich regions (TAR) within the HIV-1 gene, repressing its expression (Ou et al., 1995). Novel RNA-binding domains were identified within TDP-43, which show remarkable affinity for UG repeat motifs (Buratti and Baralle, 2001). Although normally compartmentalized within the nucleus, TDP-43 was first implicated in neurodegeneration when it was found that its presence in ubiquitin-positive cytoplasmic inclusions is a hallmark of sporadic ALS and FTD (Arai et al., 2006; Neumann et al., 2006). The cytoplasmic shift of TDP-43 has also been linked to its nuclear clearance (Mitra et al., 2019; Nana et al., 2019), resulting in multi-faceted hypotheses for its sequential loss- and gain-of-function. While its nuclear clearance has most clearly been linked to loss of splicing regulation (Donde et al., 2019; Melamed et al., 2019), increased cytoplasmic abundance has been observed to alter multiple cytoplasmic pathways. These two pathologies have reliably been observed in some CNS cells in up to 97% of all sporadic ALS cases (Ling et al., 2013). Notably, although virtually all ALS cases have TDP43 aberrant pathology in CNS cells, only a small portion of neurons within each case demonstrate this pathology.

Soon after the observation of its pathology in sporadic ALS, coding mutations in *TARDBP* were shown to be causative of ALS in a rare number of dominantly inherited cases of familial ALS, introducing a complicated framework in which TDP-43 is both a pathological hallmark of sporadic ALS and functionally related to some, but not all familial causes of ALS pathogenesis (Kabashi et al., 2008; Sreedharan et al., 2008; Pesiridis et al., 2009). Since then, novel functions of TDP-43 in numerous aspects of RNA metabolism have been identified and linked to its involvement in ALS. The following section provides focused insight into how TDP-43 and RNA interact to mediate neuronal degeneration.

### Structure of TDP-43

After the initial identification of RNA recognition motif (RRM) domains within TDP-43 (Buratti and Baralle, 2001), numerous attempts have been made to fully resolve the protein’s full-length structure. The low complexity domain of TDP-43 has significantly slowed, but not halted, progress on this front. The crystal structure of the TDP-43 RRM1 domain bound to DNA provided significant insight into its affinity for TG- and UG-rich sequences, especially when TDP-43 homodimerizes (Kuo et al., 2014), which was originally observed in previous characterizations of TDP-43 RNA targets (Tollervey et al., 2011; Xiao et al., 2011). This homodimerization depends upon intermolecular interactions between partially helical structure within the C-terminal of TDP-43 (Conicella et al., 2020). Dimerization is also dependent on the conserved E246 and D247 residues within the RRM2 domain (Shodai et al., 2012). The D247 residue is also part of a salt bridge between the two RRM domains with R151, which regulates RNA-binding function and specificity for TDP-43 (Flores et al., 2019).

Much is known about how specific point mutations confer changes to the function of TDP-43. Until recently, the lack of a high-yield protocol to produce pure, full-length TDP-43 has plagued the field. However, a major breakthrough by the Chiti group has made this possible (Vivoli Vega et al., 2019), opening the door for future investigations into the full structure and function of TDP-43 in its monomeric and homodimerized forms. Additionally, shortened isoforms of TDP-43 (sTDP-43) have been discovered in neurons that exhibit hyperactivity, further increasing the need for structural studies of full length and sTDP-43 (Weskamp et al., 2020).

### Role of TDP-43 and Its Mutant Variants in RNA Metabolism

Since TDP-43 has roles in almost every aspect of RNA metabolism (Coyne et al., 2017b), it is no surprise that its disease-related mutations have been linked to dysfunction in numerous RNA pathways. One of the first indications that the RNA-binding function of TDP-43 is altered in neurological disease came from iCLIP-RNA Sequencing experiments performed on postmortem tissue samples of FTD patients (Tollervey et al., 2011; Xiao et al., 2011). These data support altered affinity of TDP-43 for certain RNA targets, ultimately leading to altered splicing function (Tollervey et al., 2011; Xiao et al., 2011).

**FIGURE 1:**
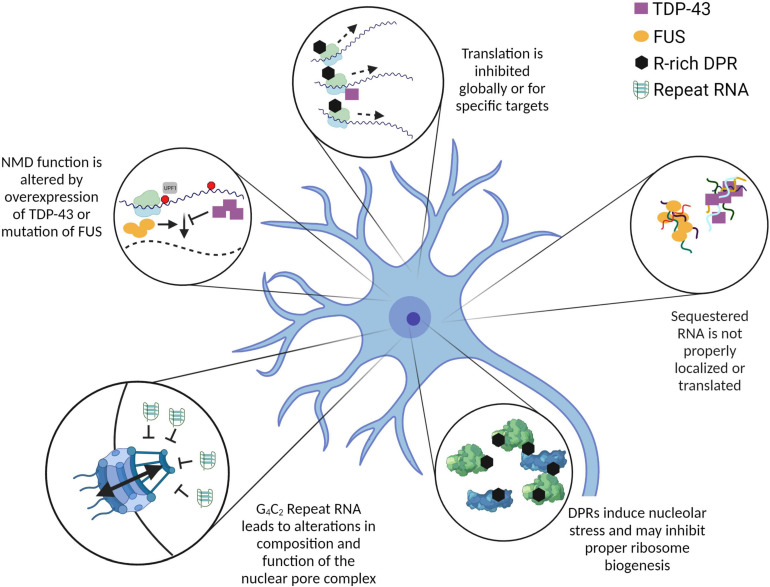
Mutations and overexpression of ALS-related proteins and RNA contribute to altered RNA metabolism. The pathways and processes depicted here are altered by the overexpression or mutation of ALS-related genes. While the mechanisms leading to these alterations are not all fully characterized, it is evident that many RNA metabolic pathways are dysregulated in the context of ALS. In some cases, such as C9ALS, RNA itself can have a significant impact on cellular homeostasis. Further investigation into the mechanistic links between these altered processes will be crucial to understand the initiating event(s) in ALS pathogenesis. Created with BioRender.com.

TDP-43 mutations were soon linked to alterations in translation of target mRNAs. In a *Drosophila* model of ALS, overexpression of wild-type and mutant TDP demonstrated loss of actively translated *futsch* mRNA (*MAP1B* in humans) (Coyne et al., 2014). The direct binding of TDP-43 to *futsch* mRNA *via* a UG-rich region within its 5′ UTR was later validated (Romano et al., 2016). This reduced translation directly leads to reduced Futsch/Map1b protein, and the destabilization of neuromuscular junctions (Coyne et al., 2014). Furthermore, it was found that *Drosophila* FMRP (FMR1 in humans) can mitigate this translational deficit and remodel RNA granules to restore the translation of Futsch/Map1b to rescue synaptic defects (Coyne et al., 2015). The same model system was used to identify Hsc70-4 (HSPA8 in humans) as an additional translational target of TDP-43 (Coyne et al., 2017a). The observed association between TDP-43 and the ribosome protein RACK1 supports the possibility that TDP-43 mutations also have a much broader propensity to alter or reduce translation (Russo et al., 2017). An independent study observed no evidence of global translation defects caused by wild-type TDP-43 or TDP-43 A315T mutant, but did provide evidence that this mutant specifically increases translation of several target mRNAs (*Camta1, Mig12*, and *Dennd4a*) (Neelagandan et al., 2019; Figure 1).

There is also evidence that specific TDP-43 mutations or overexpression may lead to alterations in non-sense-mediated decay (NMD) (Barmada et al., 2015). NMD is a translation-dependent RNA surveillance pathway that identifies and degrades mRNAs containing a premature termination codon or particularly long 3′ UTR (Kurosaki and Maquat, 2016). Notably, NMD requires an RNA-binding protein called UPF1 (Lykke-Andersen et al., 2000). In mouse primary neurons overexpressing wild-type or mutant TDP-43, UPF1 overexpression improves survival, and this rescue is depends on NMD activity of UPF1 (Barmada et al., 2015). No direct analysis of NMD function was performed in this study. However, since TDP-43 may reduce translation globally, and NMD is a translation-dependent process, it is certainly possible that a primary translation defect could lead to an apparent secondary defect in the degradation of endogenous NMD substrates (Figure 1). This lurking variable necessitates future investigations into the link between TDP-43-induced translation or splicing defects and observed alterations in downstream RNA metabolism.

Here, we only discuss RNA-binding in the context of cytoplasmic gain-of-function as it relates to TDP-43 and its disease-related mutants. However, there is powerful recent work that discusses the loss of nuclear TDP-43 function following its depletion from the nucleus (Highley et al., 2014; Donde et al., 2019; Klim et al., 2019; Melamed et al., 2019). It is important to note that while we discuss “RNA-binding” in a black-or-white manner, the full biology of TDP-43 is laden with target- and compartment-specific functions that make broad statements about its RNA-binding quite enigmatic.

### Role of RNA in TDP-43-Mediated Toxicity

In addition to the strong effect of TDP-43 on RNA metabolic processes in the cell, RNA has a robust effect on the concerted toxicity of TDP-43 in ALS (Voigt et al., 2010; Ihara et al., 2013; D’Alton et al., 2014; Kitamura et al., 2016; Chen et al., 2019). One of the first indications that RNA may mediate TDP-43 toxicity came from a targeted screen of TDP-43 variants in embryonic chick spinal cords. TDP-43 C-terminal fragment (CTF, which lacks most of RRM1 domain) and FF/LL (RNA-binding deficient) variants lead to significantly less neuronal death than WT or A315T TDP-43 when stably expressed unilaterally in *Gallus gallus* chicks (Voigt et al., 2010). An additional study in *Drosophila* demonstrated that while overexpression of WT TDP-43 in photoreceptors leads to significant cell death, removal of RRM1 or a pair of point mutations in RRM1 yield a clear reduction in toxicity (Ihara et al., 2013). Notably, this *Drosophila* model also shows that the considerable degeneration caused by ΔNLS-TDP-43 is completely mitigated by removing the RRM1 domain (Ihara et al., 2013). Together, these investigations provide powerful *in vivo* evidence that the RNA-binding function of TDP-43 may be required to induce cellular toxicity.

One finding (at first glance) may seem to contradict this hypothesis of RNA-induced toxicity. The naturally occurring C-terminal cleavage product of TDP-43 (TDP-25) leads to significantly more cell death in Neuro2A cells when compared to TDP-43, yet TDP-25 does not colocalize with RNA in cells (Kitamura et al., 2016). Thus, it is important to note at this point that toxicity resulting from overexpression of various TDP-43 species does not account for differences in their natural abundance. The C-terminal fragments of TDP-43 (TDP-25 and TDP-35) are significantly less abundant than the full-length TDP-43 (D’Alton et al., 2014). While TDP-25 overexpression may lead to cellular toxicity without the need to bind RNA, this by no means diminishes this requirement for full-length TDP-43 toxicity.

Domain-deletions provide powerful insight into the functions of TDP-43 that *potentially* lead to toxicity. Elucidating the neurodegenerating mechanism of ALS-causing mutations may be even stronger. Most TDP-43 mutations are in the C-terminal domain and do not directly interfere with RNA processing or binding functions (Chen et al., 2019). One RRM1-adjacent mutation (K181E) provides key pieces of information: K181E cannot bind RNA and leads to reduced solubility and phosphorylation of itself *and WT TDP-43* (Chen et al., 2019). As many studies have shown with overexpression and *in vitro* systems, solubility and phosphorylation of TDP-43 may be closely linked to its cytotoxicity (Arai et al., 2006; Neumann et al., 2006; Shodai et al., 2012, 2013; Sun et al., 2014; Coyne et al., 2015; Afroz et al., 2017; Li et al., 2017; Chen et al., 2019; French et al., 2019; Zacco et al., 2019). Although this review will not probe deeper into this side of TDP-43 biology, it is crucial to note that its RNA-binding function may inherently dictate its solubility.

## FUS

The *fused in sarcoma/translocated in liposarcoma* (FUS) protein was first identified as part of a fusion protein resulting from a chromosomal translocation that is common in human myxoid liposarcomas (Crozat et al., 1993). Its clear role in cancer progression led to numerous studies to understand how FUS can act as a modulator of various disease phenotypes. Importantly, the Morikawa group characterized the structure of FUS as it relates to its RNA-binding properties (Iko et al., 2004). Four years later, mutations in *FUS* were found to be causative of familial ALS[69; 70]. Since then, investigations have been focused on how these mutations alter wild-type FUS function and lead to new toxic roles for FUS in motor neurons. The following section will discuss the role of FUS mutations in altered RNA metabolism, as well as how RNA itself might mediate the conferred toxicity.

### Structure of FUS

*Fused in sarcoma/translocated in liposarcoma* is a ubiquitous RNA- and DNA-binding protein that is primarily localized to the nucleus (Crozat et al., 1993). It contains three arginine-glycine-glycine (RGG) domains, with the first two separated by an RRM domain, and the last two separated by a zinc-finger domain (Crozat et al., 1993). Due to the intrinsic disorder of the RGG domains, a full crystal structure of FUS has not yet been resolved.

Recently, the solution structure of the RRM, second RGG domain (RGG2), and the zinc-finger (ZnF) domain (amino acids 269–454) were resolved in complex with various RNA motifs (Loughlin et al., 2019). This study provided novel insights into the specificity of these three RNA-binding domains for distinct RNA motifs and secondary structures. The RRM domain was observed to bind to a broad range of RNA motifs, including a stem-loop and single-stranded RNA (ssRNA) of varying sequences. The ZnF domain bound strongly to a UGGUA motif, while exhibiting very little binding to the other tested ssRNAs. Interestingly, no significant binding occurred with RGG2 for any of the RNA motifs tested (Loughlin et al., 2019).

In the context of ALS, it is notable that none of the FUS mutations identified in ALS patients are within the RRM or ZnF domains. One explanation is that mutations within these domains are physiologically silent while alternatively they may be lethal. FUS is non-essential in murine (Kino et al., 2015) and human cell (Reber et al., 2018) models. Thus, if mutations within these domains are lethal, it is unlikely that this lethality is due to loss of normal FUS function. Instead, it is possible that such mutations lead to aberrant (and lethal) function, perhaps *via* promiscuous RNA-binding. Indeed, a triplet of K→A mutations within the RRM lead to a loss of FUS-ΔNLS ability to compartmentalize itself within either the nucleus or cytoplasm (Liu et al., 2013). It is unknown how these mutations affect the function of full-length FUS.

As more complete structures of FUS become resolved, it will be crucial to review our current understanding of FUS structural mutations as they relate to ALS pathogenesis.

### Role of FUS Mutations in RNA Metabolism

As would be expected of an RNA-binding protein, many of the ALS associated FUS mutations alter the protein’s function within various RNA metabolism pathways. When mutations in FUS were first identified to cause ALS, the main observation involved these mutants’ mislocalization to the cytoplasm (Kwiatkowski et al., 2009; Vance et al., 2009). Since then, a significant portion of the ALS-associated FUS mutations have been observed within the nuclear localization signal (NLS), residues 510–526 (Shang and Huang, 2016).

*Fused in sarcoma/translocated in liposarcoma* P525L has been shown to localize in large cytoplasmic accumulations when overexpressed in SH-SY5Y cells, and poly-A mRNA is enriched in these accumulations (Takanashi and Yamaguchi, 2014; Figure 1). However, the authors establish no causal link between this observation and potentially altered RNA metabolism. Theoretically, the mRNA present within these accumulations would be resistant to degradation by typical surveillance pathways. While this may shift the global transcriptome by stabilizing a subset of mRNA, it is unclear how this may functionally affect the proteome, as these mRNAs may not be translated while they are sequestered.

Kinesin-1 (*Kif5b*) mRNA and protein are particularly enriched in FUS inclusions (Yasuda et al., 2017). This leads to a deficit in the ability of kinesin-1 to transport specific mRNAs *to* neuronal axons (Yasuda et al., 2017). Although the authors provide no experimental connection between mis-localization of kinesin-1 target mRNAs and known ALS pathogenesis, it is possible that hereunto unidentified mRNAs are similarly mislocalized, contributing directly to neuronal degeneration. Kinesin-1 also has a role in transporting materials anterogradely along axons (Lim et al., 2017), though it is not yet known whether FUS inclusions modulate this particular function of kinesin-1. Interestingly, mutations in *KIF5A*, another gene encoding a kinesin protein, have recently been shown to cause ALS (Brenner et al., 2018; Nicolas et al., 2018). This warrants additional investigations into the possible connection between FUS and KIF5A in the context of ALS.

Whereas previous reports indicate reduced NMD activity in TDP-43 models of ALS (Barmada et al., 2015), FUS models hint at potentially increased NMD activity. Fibroblasts from patients with mutations in FUS have relatively high levels of UPF1 (Kamelgarn et al., 2018). This result, in addition to the reduced expression of several NMD reporters, is interpreted to indicate an increase in the activity of the NMD pathway. The authors present a model of mutant FUS-disrupted autoregulation within the NMD pathway (Kamelgarn et al., 2018; Figure 1). However, further investigation is needed to reconcile the apparent disconnect between the observed reduction in translation and increase in NMD activity caused by FUS mutations.

### Role of RNA in FUS-Mediated Toxicity

Amyotrophic lateral sclerosis-linked FUS mutations clearly alter RNA metabolism, but this influence is not a one-way street. RNA itself can have a profound impact on FUS-mediated cellular toxicity. The study of FUS mutants with reduced or absent RNA-binding ability has provided a wealth of information about how RNA can mitigate, or instigate, toxicity.

*Fused in sarcoma/translocated in liposarcoma* has been shown to bind RNA *via* two different types of interaction: a monomer binding tightly to ssRNA, and a pair of monomers binding weakly (Niaki et al., 2020). This binding depends on the length of the RNA, and is significantly altered by mutations in FUS arginine residues (R244, R216, and R514), contributing to these mutants’ propensity to form larger droplets than FUS WT (Niaki et al., 2020). Interestingly, the ubiquilin-like protein ubiquilin-2 is able to modify the FUS-RNA interaction, reducing its tendency to form large liquid droplets *in vitro* or stress granules in cells (Alexander et al., 2018). No direct assay was performed to determine if these larger droplets may reduce survival or lead to cellular toxicity. *In vitro*, RNA has the effect of reducing FUS liquid-liquid phase separation (LLPS) and helps maintain FUS solubility in the nucleus (Monahan et al., 2017; Maharana et al., 2018).

Expression of cytoplasmic FUS that lacks the RRM in mice leads to early death, although these mice don’t exhibit signs of significant neurodegeneration, despite the presence of FUS inclusions in the cortex and brainstem (Robinson et al., 2015), and thus the actual mechanism underlying death is not known and could involve non-CNS tissues in this mouse model. This is not unprecedented, as early mutant TDP-43 mouse models died as a result of gastrointestinal problems, (not seen in the true human disease) rather than CNS degeneration (Hatzipetros et al., 2014).

While there is clear evidence *in vitro* that loss of the FUS-RNA interaction can lead to formation of larger aggregates or inclusions, it is not fully clear that this has any neuronal-specific consequence *in vivo*. In order to truly understand whether FUS-RNA binding is a significant contributor to ALS pathogenesis, it may be necessary to use *in vivo* knock-in studies to perform physiologically relevant experiments (Lopez-Erauskin et al., 2018; Reber et al., 2018).

## C9ORF72

### Introduction

Although many of the genes linked to ALS demonstrate disease-causing point mutations, the *C9ORF72* mutation exists as a GGGGCC hexanucleotide repeat expansion (HRE) within the first intron of the gene (DeJesus-Hernandez et al., 2011; Renton et al., 2011). This mutation accounts for the largest cohort of both familial (20–50%) and sporadic (5–20%) ALS (sALS) cases (collectively referred to as C9ALS) (Ling et al., 2013). In affected individuals, the HRE may be thousands of repeats long, but can also exhibit intermediate toxicity with as few as 24 repeats (Iacoangeli et al., 2019). Notably, this expansion ranges from 0 to 24 repeats in unaffected individuals. The HRE leads to three primary changes in neurons, each of which may contribute to the overall toxicity caused by the mutation.

First, the HRE reduces the abundance of *C9ORF72* mRNA (DeJesus-Hernandez et al., 2011) and C9ORF72 protein (Renton et al., 2011; Waite et al., 2014). *C9ORF72* encodes C9ORF72, a protein which helps regulate autophagy (Webster et al., 2018). Therefore, it has been hypothesized that haploinsufficiency of C9ORF72 reduces autophagic regulation. Notably, heterozygous loss of the protein/mRNA, comparable to human disease has no overt pathological toxicity, although subtle changes in microglial RNA have been detected. In that regard, an increase in autoimmune diseases have been seen a very small number of C9ALS/FTD patients—but to emphasize, C9ALS is not a disorder with prominent autoimmune disease burden. However, complete loss of the protein/RNA, not seen in human C9ALS, leads to a severe autoimmune dysfunction in rodent, not typical of C9ALS, or ALS in general (Ciura et al., 2013; Shi et al., 2018).

Second, the HRE-containing RNA, though present within an intron, translocates to the cytoplasm (Donnelly et al., 2013; Lagier-Tourenne et al., 2013). Here, it may aberrantly sequester miscellaneous proteins, diminishing their ability to function normally. Notably, the antisense repeat RNA (CCCCGG) may also be transcribed and translocated to the cytoplasm (Gendron et al., 2013).

Third, cytoplasmic repeat RNA can assume highly-structured forms (Fratta et al., 2012) which can recruit ribosomal machinery to initiate translation. *Via* this repeat-associated non-ATG (RAN) translation, ribosomes bind to the sense and antisense repeat RNA and elongate to produce dipeptide repeat (DPR) proteins[5; 96]. Owing to ribosomal frameshifting, two unique DPRs are produced from each HRE RNA strand, with a common DPR expressed by both [GGGGCC RNA yields poly(GA) and poly(GR), CCCCGG RNA produces poly(PA) and poly(PR), both strands yield poly(GP)] (Ash et al., 2013; Gendron et al., 2013; Zu et al., 2013; Tabet et al., 2018). These five DPRs have been hypothesized to induce toxicity through a plethora of mechanisms.

Due to the complex nature of this mutation, as well as the focus of this review, the following discussion will be limited to an assessment of how the HRE RNAs and DPRs alter RNA processing pathways, as well as how the repeat RNA itself induces neuronal toxicity.

### Structure of C9ORF72 HRE RNA and DPRs

The GGGGCC HRE RNA has been shown to form G-quadruplexes *in vitro* (Fratta et al., 2012; Reddy et al., 2013; Conlon et al., 2016), and these structures have been observed in the postmortem tissue of ALS patients (Conlon et al., 2016). Few studies have delved into the structure of purified (GGGGCC)_n_ RNA under various conditions (Dodd et al., 2016; Mulholland et al., 2020), though the physiological structure of this RNA remains elusive. It is known that some subset of the HRE RNA is present in the form of foci, which each contain a single highly structured RNA molecule (Liu et al., 2017), though studies of the structures that soluble HRE RNA adopts have yet to be performed. There remains an unmet need to understand the various states in which the HRE RNA exists *in vivo*. Future investigations may need to probe the distinct roles of soluble (unstructured) and insoluble (highly structured) HRE RNA in conferring cellular toxicity.

Due to the variable length of the five DPRs, as well as their relative insolubility when purified, it has been difficult for investigators to derive their crystal or nuclear magnetic resonance structures. However, some headway has been made using techniques like circular dichroism to elucidate the relatively disordered secondary structure of the DPRs (Flores et al., 2016). To date the relative amounts of the individual repeat polypeptides and the true range of their lengths *in vivo* remain unknown.

Clearly, the field of investigating the HRE RNA and DPR structures is wide open. Advancements therein will provide extremely useful data to inform future investigations into their toxic role in disease.

### Role of the C9ORF72 Mutation in RNA Metabolism

The first indication that the HRE RNA may contribute to alterations in RNA metabolism came with the observations that the HRE foci (punctate inclusions containing the HRE sense and antisense RNA) sequester various RNA-binding proteins (RBPs) (Donnelly et al., 2013; Lee et al., 2013; Cooper-Knock et al., 2014; Conlon et al., 2016). The hypothesis presented is that “locking up” of RBPs reduces their endogenous functions to maintain RNA homeostasis; this is a common feature among selected neurological/neuromuscular diseases having been previously well-studied in myotonic dystrophy (Jiang et al., 2004).

Dipeptide repeats have also been shown to interact with RBPs, altering their endogenous functions. Co-immunoprecipitation of synthetic poly(PR) after incubation with NSC34 cells yielded a variety of poly(PR)-interacting proteins that regulate translation and ribosome assembly (Kanekura et al., 2016). Independent screens also identify RBPs that are significantly altered in function by the presence of high poly(PR) and poly(GR) concentrations (Hartmann et al., 2018; Kramer et al., 2018; Zhang et al., 2018). These findings are strongly validated with the observation that the presence of high poly(PR) concentrations significantly reduced association of the crucial translation initiation factors eIF4E and eIF4G with poly(A) RNA (Kanekura et al., 2016). Additional experiments show that high poly(PR) or poly(GR) concentrations (*via* overexpression or exogenous addition) reduce translation (Hartmann et al., 2018; Zhang et al., 2018; Sun et al., 2020). Indeed, controlled titration of synthetic poly(PR)_20_ shows almost perfect alignment with cycloheximide in terms of the effect on reducing translation (Sun et al., 2020; Figure 1).

The potent effect of poly(GR) and poly(PR) on translation becomes quite relevant with the “apparent” discovery of NMD defects in C9ALS. Multiple recent studies have proposed that NMD function is altered by the HRE, whether it be due to DPR toxicity or HRE RNA (Xu et al., 2019; Ortega et al., 2020; Sun et al., 2020). Notably, the observations of NMD dysfunction provided by these studies are either unconvincing (derived from minor phenotypes with a very low sample size) (Ortega et al., 2020) or stem from significant translation defects induced by overexpression of arginine-rich DPRs (Xu et al., 2019; Sun et al., 2020). Thus, it is difficult to conclude from these studies whether NMD function is at all altered by the HRE in human patients. A very new study has attempted to answer this question using an human induced pluripotent stem cell-derived spinal neuron (iPSN) model of C9ALS (Zaepfel et al., 2021). Following validation of several bona fide transcripts as substrates for NMD, it was demonstrated that neither the steady-state abundance nor stability of these substrates is altered in C9ALS patient-derived iPSNs relative to age- and sex-matched controls (Zaepfel et al., 2021). Although this strongly refutes the presence of any NMD dysfunction in C9ALS, this study corroborates that UPF1 is neuroprotective in this context (Zaepfel et al., 2021). Evidence is provided that UPF1 overexpression reduces the abundance of DPRs in multiple models, potentially explaining its neuroprotective effects (Zaepfel et al., 2021). However, much is still left unanswered by these papers to precisely explain the role that UPF1 plays in supporting neuronal health.

In addition to the strong RBP sequestration caused by the HRE RNA and DPRs, there is also evidence that the biogenesis of ribosomes may be disrupted by this mutation. Poly(GR) and poly(PR) colocalize with ribosomal proteins in C9ALS patient tissue (Hartmann et al., 2018), which hints at a possible unbalancing of the carefully maintained stoichiometry of ribosomal proteins with ribosomal RNA. When overexpressed, poly(PR) and poly(GR) both flock to nucleoli, the ribosome-generating centers of the cell (Tao et al., 2015). This localization of arginine-rich DPRs to the nucleoli leads to nucleolar stress (Tao et al., 2015; Mizielinska et al., 2017; Hartmann et al., 2018), which may lead to significant alterations in the proper assembly of ribosomes themselves (Yang et al., 2018). There exists a possibility that this nucleolar stress leads to mis-assembly of ribosomes, which may compound the reduced translation noted above.

### Role of RNA in C9ORF72 HRE-Mediated Toxicity

Relative to the plethora of investigations designed to understand the contribution of DPRs to neuronal toxicity, little is known about if and how the HRE RNA itself leads to disease phenotypes. This is due, in large part, to early *Drosophila* models that overexpress the HRE RNA or GGGGCC RNA that contains intermittent stop codons (“RNA only”) (Mizielinska et al., 2014). While the former demonstrates production of both the repeat RNA and DPRs, RNA only flies produce only the RNA. These models, taken at face value, provided a controlled opportunity to determine the respective contributions of these two sets of toxic products. Indeed, severe degenerative phenotypes were observed in flies expressing the uninterrupted repeat, but flies expressing only the repeat RNA showed no signs of toxicity, despite containing repeat RNA foci (Mizielinska et al., 2014). This observation quite resoundingly turned most of the ALS field away from investigations of potential RNA-contributed toxicity. To many, elucidating a role for repeat RNA in HRE-mediated degeneration seemed like a proverbial search for a needle in a haystack without knowing that the needle exists.

This needle was recently found during a journey to understand the initiating event for nucleocytoplasmic transport dysfunction in C9ALS. Many labs have reported that neurons harboring the HRE demonstrate reduced ability to regulate transport across the nuclear pore complex (Freibaum et al., 2015; Zhang et al., 2015). For many years, little to no headway was made in identifying the root cause of this dysfunction. Then a comprehensive investigation identified the precise nucleoporins (the proteins that assemble into the nuclear pore complex) that are absent in C9ALSpatient neurons (Coyne et al., 2020). One such nucleoporin, Pom121, is proposed as the initial domino in a series of lost nucleoporins (Coyne et al., 2020). A painstaking number of orthogonal hypothesis-killing experiments brings the authors to a unified conclusion: the entirety of nuclear pore complex disassembly is instigated by the HRE RNA, not DPRs or loss of C9ORF72 protein (Coyne et al., 2020; Figure 1). The precise mechanism of HRE RNA-mediated disassembly of the nuclear pore complex remains enigmatic. Why, then, did that original *Drosophila* model fail to demonstrate RNA-induced toxicity? The answer is simple: Pom121 is not conserved in fruit flies (Coyne et al., 2020). Without the presence of this initial domino, the rest do not fall.

### RNA-Based Therapies for ALS

RNA-centric therapies for ALS are quite varied, due to the distinct roles that RNA plays in instigating or ameliorating toxicity in different models as described above. In the case of C9ALS, antisense oligonucleotides (ASOs) have been utilized widely in the laboratory and clinical settings. ASOs are heavily modified DNA sequences that are designed complimentary to a target RNA. Upon annealing, they induce RNase H-dependent degradation of their target while the ASOs themselves remain intact (Ly and Miller, 2018). ASOs against the *C9ORF72* HRE RNA, though initially developed as a tool to eliminate RNA foci in cultured cells (Donnelly et al., 2013; Lagier-Tourenne et al., 2013), have been developed for trials in ALS patients, although the results of this novel intervention are as yet unknown. Since these initial ASOs targeted the sense strand repeat-containing introns of *C9ORF72* RNA, the prediction is that pathogenic RNA will be degraded without affecting the abundance of coding mRNA. Whether ASO targeting the antisense strand may also be necessary for disease mitigation is unknown—but should be investigated at least preclinically.

Targeted knockdown of TDP-43 and FUS, however, may not be as useful. While it is a valid hypothesis that reducing their abundance would decrease their propensity for pathogenesis, both TDP-43 and FUS have important biological functions. Knocking down these mRNAs may open a whole new can of worms, where loss of their endogenous function may be just as toxic as their disease-related gain of function. Numerous *in vitro* and *in vivo* studies have clearly demonstrated that simple knockdown or overexpression of wild type TDP-43 is terribly cytotoxic. One workaround that may prove effective in the realm of TDP-43 pathogenesis comes with the use of bait oligonucleotides (Mann et al., 2019). Introduction of TDP-43-targeted RNA oligonucleotides to cells overexpressing TDP-43 leads to decreased neurotoxicity, hypothetically by reducing the aberrant phase transition of TDP-43 (Mann et al., 2019). Although this study erroneously counts individual cells as independent samples, the effect of this method of treatment is notable (Mann et al., 2019).

Targeting downstream molecular events that follow TDP-43 loss of function, such as Stathmin2 mis-splicing, are open to candidate therapies and may allow at least some mitigation of TDP-43 loss-of-function toxicity (Melamed et al., 2019). Finally, understanding the mechanism that lies upstream of aberrant TDP-43 loss of nuclear localization may prove to be the most encompassing approach to therapy—not only for ALS—but the other disorders of TDP-43 pathology.

## Conclusion

Decades of research and independent investigations have provided clear evidence that RNA biology is commonly disrupted in the context of ALS. Yet our understanding of RNA metabolism alterations caused by each mutation lacks a unifying link. Indeed, the missing link may be found by investigating RNA metabolism in sALS, where the vast majority of cases are caused by hitherto unknown mechanisms. If a prominent, causative mechanism for sALS pathogenesis is identified within the realm of RNA biology, only then can the field clarify the bidirectional role for RNA in ALS neurotoxicity and neuroprotection.

## Author Contributions

BLZ wrote the manuscript. JDR edited the manuscript. Both authors contributed to the article and approved the submitted version.

## Conflict of Interest

The authors declare that the research was conducted in the absence of any commercial or financial relationships that could be construed as a potential conflict of interest.
